# Comparison of multiple treatment regimens in children with *Helicobacter pylori* infection: A network meta-analysis

**DOI:** 10.3389/fcimb.2023.1068809

**Published:** 2023-02-23

**Authors:** Miaomiao Liang, Chengbi Zhu, Peipei Zhao, Xiaohui Zhu, Junwei Shi, Bin Yuan

**Affiliations:** ^1^ Department of Pediatrics, Affiliated Hospital of Nanjing University of Chinese Medicine, Nanjing, China; ^2^ Jiangsu Key Laboratory of Pediatric Respiratory Disease, Institute of Pediatrics, Medical Metabolomics Center, Nanjing University of Chinese Medicine, Nanjing, China

**Keywords:** *Helicobacter pylori*, children, treatment regimens, efficacy, network meta-analysis

## Abstract

**Background:**

Multiple regimens have been widely used in the eradication treatment of *Helicobacter pylori* infection in children. However, there is a lack of comparison and evaluation of their effectiveness in different regions of the world.

**Methods:**

Randomized controlled trials were retrieved. Review Manager 5.4, Stata SE 15 and R 4.0.4 statistical software were used to analyze date. The ranking probability is assessed according to the surfaces under cumulative ranking (SUCRA).

**Results:**

163 studies were eligible for this study, involving 336 arms and 18,257 children, and 10 different interventions. The results showed that the eradication rates of sequential therapy with probiotics (SP), bismuth-containing quadruple (Quadruple) therapy, concomitant therapy and PCN therapy were at least 90%. Cumulative ranking showed that SP therapy had the best eradication effect (SUCRA 92.7%) whereas Bismuth-containing triple therapy (B) had the worst (SUCRA 3.5%). Subgroup analysis suggested that SP therapy ranked first in China and other regions, and the ranking of Triple therapy with probiotics therapy (TP) was equally stable (SUCRA 72.0% *vs* 76.4% respectively). The security of the SP and TP therapy had great advantages.

**Conclusions:**

As for the eradication treatment of *Helicobacter pylori* infection in children, SP therapy ranks highest. SP and TP therapies are most safe.

## 1 Introduction

*Helicobacter pylori* infection in children is a common pediatric disease. Although an epidemiology investigation has showed that the prevalence of *H. pylori* is decreasing in developed countries (Burucoa and Axon, 2017), it remains a serious problem in developing and less developed regions (Daugule et al., 2016; Hooi et al., 2017). *H. pylori* was discovered by Robin Warren and Barry Marshall during the study of gastric biopsy specimens in 1982 (Goodwin et al., 1989). It usually parasitizes in the human gastric mucosa and spreads through the oral-oral and fecal-oral routes. Most children infected with the bacteria are asymptomatic or show mild symptoms, such as common regular epigastric pain, loss of appetite, nausea and vomiting. Thereby they are often easily overlooked by their parents, and the disease is aggravated without timely diagnosis and treatment. A few patients with *H. pylori* infection have symptoms of recurrent headache (Shivalingaiah et al., 2019; Cavestro et al., 2022). Severe cases may also suffer from gastritis, peptic ulcer, gastric mucosa-associated lymphoid tissue (MALT) lymphoma, and even gastric cancer (Plummer et al., 2015), which have a serious impact on children’s quality of life and growth. *H. pylori* gastritis was officially recognized as an infectious disease in 2015 (Sugano et al., 2015). Compared with adults, children have poorer digestive tract mucosal resistance and self-repair function, so the gastric mucosal damage caused by *H. pylori* infection is more serious, and the clinical symptoms are often more obvious. Therefore, children from families with low economic level and poor living environment often become a high-risk group of *H. pylori* infection (Czinn, 2005).

The North American Society for Pediatric Gastroenterology, Hepatology, and Nutrition (NASPGHAN) and the European Society for Pediatric Gastroenterology, Hepatology, and Nutrition (ESPGHAN) recommend in their guidelines for the treatment of children and adolescents that a 14-day triple therapy of different antibiotics should be developed for children infected with *H. pylori* according to the results of drug sensitivity test, while children with unknown antibiotic sensitivity were given 14 days of high-dose triple therapy or bismuth-containing quadruple therapy (Jones et al., 2017). However, with the abuse of antibiotics and the enhancement of drug resistance, the effect of simple standard triple therapy eradicating *H. pylori* is on the wane, and the recurrence rate of *H. pylori* also has showed an upward trend in recent years (Zhao et al., 2021). This has become a great challenge for clinicians. With the continuous exploration of the disease, a variety of eradication methods have been introduced, including metronidazole replacement triple therapy, bismuth triple therapy (excluding PPI) and quadruple therapy, probiotic quadruple therapy, sequential therapy, concomitant therapy (Dong and Zhang, 2022). Nevertheless, most of them are empirical treatments in clinical application, and there is no unified drug use standard for children up to now. At present, evaluating the eradication rate of each treatment mostly relies on RCT and pairwise meta-analysis (Zhou et al., 2016), and there is a lack of comprehensive comparison of their efficacy. Compared with traditional meta-analysis, network meta-analysis (NMA) can collect a wide variety of interventions under the same conditions and rank them according to their efficacy (Liu et al., 2014). The purpose of this study is to summarize and compare the eradication rate and safety of treatments of *H. pylori* in children around the world through NMA, hoping to provide reference for future guidelines and clinical medication for the treatment of *H. pylori* infection in children.

## 2 Materials and methods

The protocol for this Network Meta-analysis was prospectively registered with PROSPERO (CRD42022342787).

### 2.1 Inclusion criteria

#### 2.1.1 Object

*H. pylori* infection was diagnosed by histopathological examination, urea breath test (UBT), fecal HP antigen test (SAT), or rapid urease test; the age was less than 18 years old; there was no restriction in race, sex, and course of disease; patients had no other serious gastrointestinal diseases; the subjects did not use related antibiotics to treat other diseases at the same time.

#### 2.1.2 Type

Eligible studies were randomized controlled trials (RCTs). The language was limited to Chinese and English.

#### 2.1.3 Interventions

All the children were randomly assigned to at least 2 groups. The intervention measures in each group were in line with the standards of the guidelines, and the course of treatment was at least 7 days.

#### 2.1.4 Outcomes

The eradication rate reexamined after at least 2 weeks of treatment was used as the outcome index of effectiveness, and the total side effects rate was used as the safety evaluation index.

### 2.2 Exclusion criteria

Retrospective studies, cohort studies, case reports, commentaries, conference abstracts, animal experiments, reviews, and repeatedly published articles; articles with inappropriate intervention measures, such as RCTs in which the two groups have the same regimen but different doses or courses of treatment; articles with unavailable or missing data; articles for which outcome measures can’t be combined.

### 2.3 Publication search strategy

Relevant trials were searched in 8 databases, including PubMed, Embase, Web of Science, the Cochrane Library, China National Knowledge Infrastructure (CNKI), Chinese Biomedical Literature database (CBM), Wanfang Database, and VIP Information (VIP). The following search phrases were used: “Helicobacter pylori”, “Campylobacter pylori”, “children”, “randomized controlled trial” and so on. If there are duplicate publications, only the one published earlier will be retained. In order to ensure the comprehensiveness of the search results, the references in important articles were screened manually. The specific retrieval methods are shown in **Supplementary Table S1**.

### 2.4 Literature screening, data extraction and quality evaluation

Two researchers (LMM, ZCB) independently screened and cross checked the literature. Any dissents were solved by discussing together or consulting a third researcher for assistance. All the searched articles were imported into EndnoteX9.1 for management. The extracted data include the first author, year of publication, country, total sample size, study design, regimens compared, course of treatment. The data were imported into the Excel2007 and cross-checked by the aforesaid two researchers. According to the bias risk assessment scale of randomized controlled trials recommended by Cochrane system evaluator manual 5.1.0, the bias risk quality assessment tool with 6 items in ReviewManager5.4 was used to evaluate the quality of included literature (Cumpston et al., 2019).

### 2.5 Statistical analysis

Stata SE 15 was used for data processing and a random-effects model was used to draw a network evidence diagram. When there was a closed loop, a heterogeneity test was further performed for both direct evidence and indirect evidence. A P > 0.05 indicated a good consistency and therefore a consistency model was applied. The gemtc program package called by R 4.0.4 statistical software and the Markov Chain Monte Carlo were used for Bayesian network meta-analysis. The Markov chain was set to 4 with iterations set to 50,000 and annealing set to 20,000, in order to eliminate the influence of the initial values. The outcome measures in the present study were binary variables. Therefore, the odds ratio (OR) with 95% confidence intervals (CI) was used as the effect size of this statistical analysis. When P < 0.05, the difference was statistically significant. To explore a more effective and safer treatment, Stata SE 15 was also used to calculate and visualize the surface under the cumulative ranking (SUCRA) of the cumulative probability according to the matrix table of ranking probability obtained from R 4.0.4 software. The larger the SUCRA value was, the more effective and safer the treatment was. And then, the difference of eradication rate between different regions was examined by subgroup analysis. Finally, an inverted funnel plot was drawn to assess publication bias and determine whether publication bias or small-sample effects existed (Dias et al., 2010).

## 3 Results

### 3.1 Literature screening

**Figure 1:**
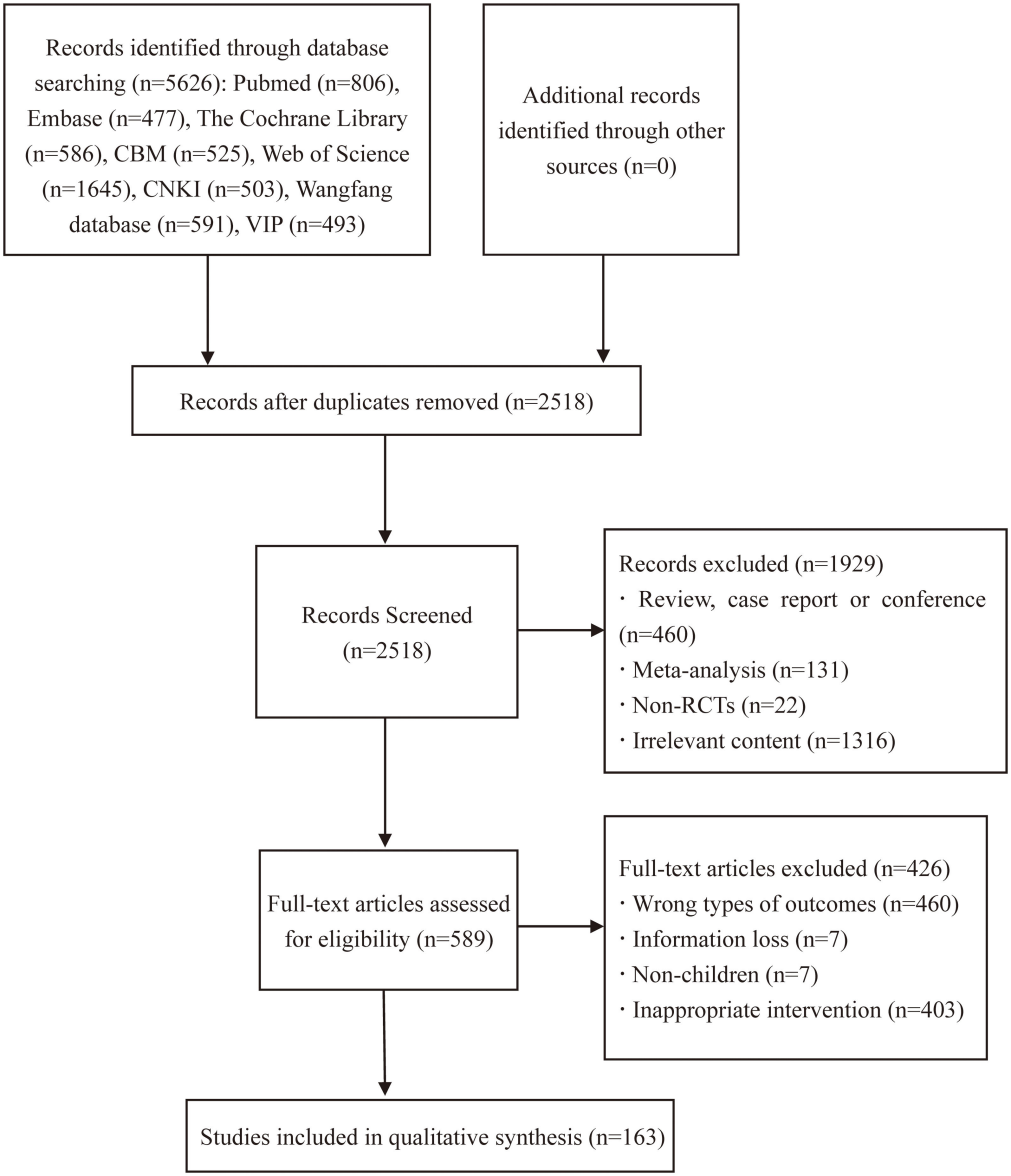
Flow diagram of the study search process.

Initially 5626 related articles were searched and strictly screened in Endnote X9.1 software. After the removal of 3108 duplicates, 1929 articles were excluded based on topics and abstracts. After full-text review, 426 articles were excluded. Finally, 163 articles (Li, 2003; Francavilla et al., 2005; Sýkora et al., 2005; Bahremand et al., 2006; Goldman et al., 2006; Lionetti et al., 2006; Huang, 2007; Tang, 2008; Wan and Liao, 2008; Hurduc et al., 2009; Lei, 2009; Li, 2009; Worona-Dibner, 2009; Xiong et al., 2009; Zhang, 2009; Li, 2010; Liu, 2010; Pan et al., 2010; Zhu, 2010; Albrecht et al., 2011; Bontems et al., 2011; Li and Yu, 2011; Li et al., 2011; Liu F. et al., 2011; Liu Y. et al., 2011; Wang et al., 2011; Wu, 2011; Chu, 2012; Ji, 2012; Li and Lin, 2012; Lie, 2012; Liu, 2012; Rong, 2012; Tolone et al., 2012; Wu et al., 2012; Zhang, 2012; Zhang and Li, 2012; Ahmad et al., 2013; Baysoy et al., 2013; Chen, 2013; Chen et al., 2013; Huang et al., 2013; Laving et al., 2013; Pan, 2013; Sun et al., 2013; Xu et al., 2013; Yang et al., 2013; Zhang, 2013; Zhao et al., 2013; Zhong, 2013; Chen et al., 2014; He, 2014; Hou, 2014; Ke, 2014; Ku et al., 2014; Kutluk et al., 2014; Li and Zhou, 2014; Liu, 2014; Wang, 2014; Wang and Feng, 2014; Wang and Huang, 2014; Yang, 2014; Akcam et al., 2015; Chen, 2015; Gao et al., 2015; Islek et al., 2015; Liu et al., 2015; Luo et al., 2015; Sun, 2015; Wang and Liu, 2015; Xiao et al., 2015; Zhang, 2015a; Zhang, 2015b; Zhang, 2015c; Zhang, 2015d; Zhao and Zhou, 2015; Zhong et al., 2015; Zhou and Li, 2015; Zhou and Li, 2015; Cong and Hu, 2016; Fan et al., 2016; Gong, 2016; Han, 2016; Huang, 2016; Li et al., 2016; Qin, 2016; Shahraki et al., 2016; Xu et al., 2016; Zhang, 2016; Zhou, 2016; Zhu and Li, 2016; Fang and Li, 2017; He, 2017; Li, 2017; Tang and Liu, 2017; Urganci, 2017; Ustundag et al., 2017; Wang, 2017; Wu, 2017; Xiang et al., 2017; Zhu et al., 2017; Chen et al., 2018; Fan, 2018; Han, 2018; Huang, 2018; Li, 2018; Li and Shi, 2018; Li J., 2018; Li, 2018; Luo, 2018; Qiu et al., 2018; Wu et al., 2018; Xie, 2018; Zhang, 2018; Zhou, 2018; Zhou and Shi, 2018; He et al., 2019; He et al., 2019; Lhamo and Tinley, 2019; Liang, 2019; Lin and Huang, 2018; Liu and Fang, 2019; Liu et al., 2019; Mei et al., 2019; Wang, 2019; Wang et al., 2019; Zhang, 2019; Zhang et al., 2019b; Zhang et al., 2019a; Zhao, 2019; Zhou et al., 2019; Zhu, 2019; Chen et al., 2020; Chen X. et al., 2020; Lai and Liang, 2020; Li, 2020; Liu, 2020; Liu and Zhang, 2020; Rong and Xiao, 2020; Su, 2020; Sun et al., 2020; Yang et al., 2020; Yao and Luo, 2020; Zhao, 2020; Zheng et al., 2020; Gao, 2021; Han, 2021; Hu, 2021; Ke et al., 2021; Li, 2021a; Li, 2021b; Li and Qi, 2021; Liu, 2021; Liu and Wu, 2021; Shi et al., 2021; Xiao and Shao, 2021; Yang, 2021; Yuan, 2021; Yuan and Ning, 2021; Zhang, 2021; Zhou, 2021; Liu et al., 2022; Zhou, 2022) were adopted, including 21 English articles and 142 Chinese studies. The detailed search strategy is described in **Figure 1**.

### 3.2 Basic characteristics

163 studies were included, with a total of 336 therapeutic arms, including 156 dual arm trials, 4 three-arm trials (Zhang, 2009; Liu et al., 2011; Li, 2017; Luo, 2018) and 3 four-arm trials (Rong, 2012; Zhu et al., 2017; Chen et al., 2020). The total sample size is 18257 cases, with the largest sample size of 541 cases and the minimum sample size of 15 cases of per arm. The 10 treatment regimens implemented by these researchers and the number of subjects around the world are as follows (in alphabetical order):

1. B (Bismuth-containing triple therapy), n=348;
2. Concomitant (Concomitant therapy), n=50;
3. PAC (PPI, amoxicillin and clarithromycin), n=7448;
4. PAF (PPI, amoxicillin and nitrofuran drugs), n=196;
5. PAN (PPI, amoxicillin and nitroimidazoles), n=2291;
6. PCN (PPI, clarithromycin and nitroimidazoles), n=908;
7. Quadruple (Bismuth-containing quadruple therapy), n=1537;
8. Sequential (Sequential therapy), n=1964;
9. SP (Sequential therapy with probiotics), n=189;
10. TP (Triple therapy with probiotics), n=3326.

Basic information is provided in **Supplementary Table 2**.

### 3.3 Risk assessment of bias

**Figure 2:**
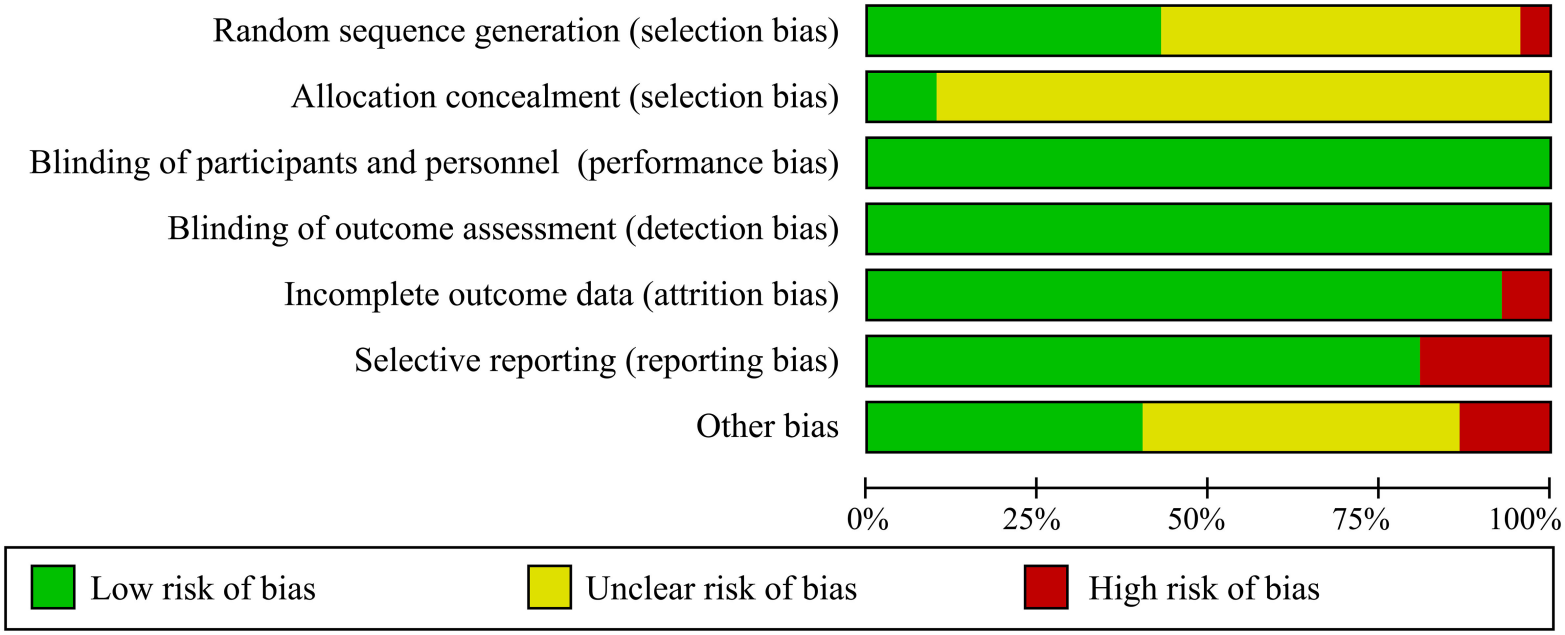
Risk of bias graph of methodological bias.

All studies mentioned randomness in the allocation, but some were considered “unclear” or “high risk” for unspecific allocation schemes or nonstandard allocation schemes. Eradication rate is an objective detection index and does not represent the subjective will of researchers. Therefore, performance bias and detection bias were regarded as “low risk”. Any study with drop-out cases is considered as “high risk” in selective reporting. In most comparisons, the certainty of the evidence was moderate. The overall evaluation results are shown in **Figure 2**.

### 3.4 Network map

**Figure 3:**
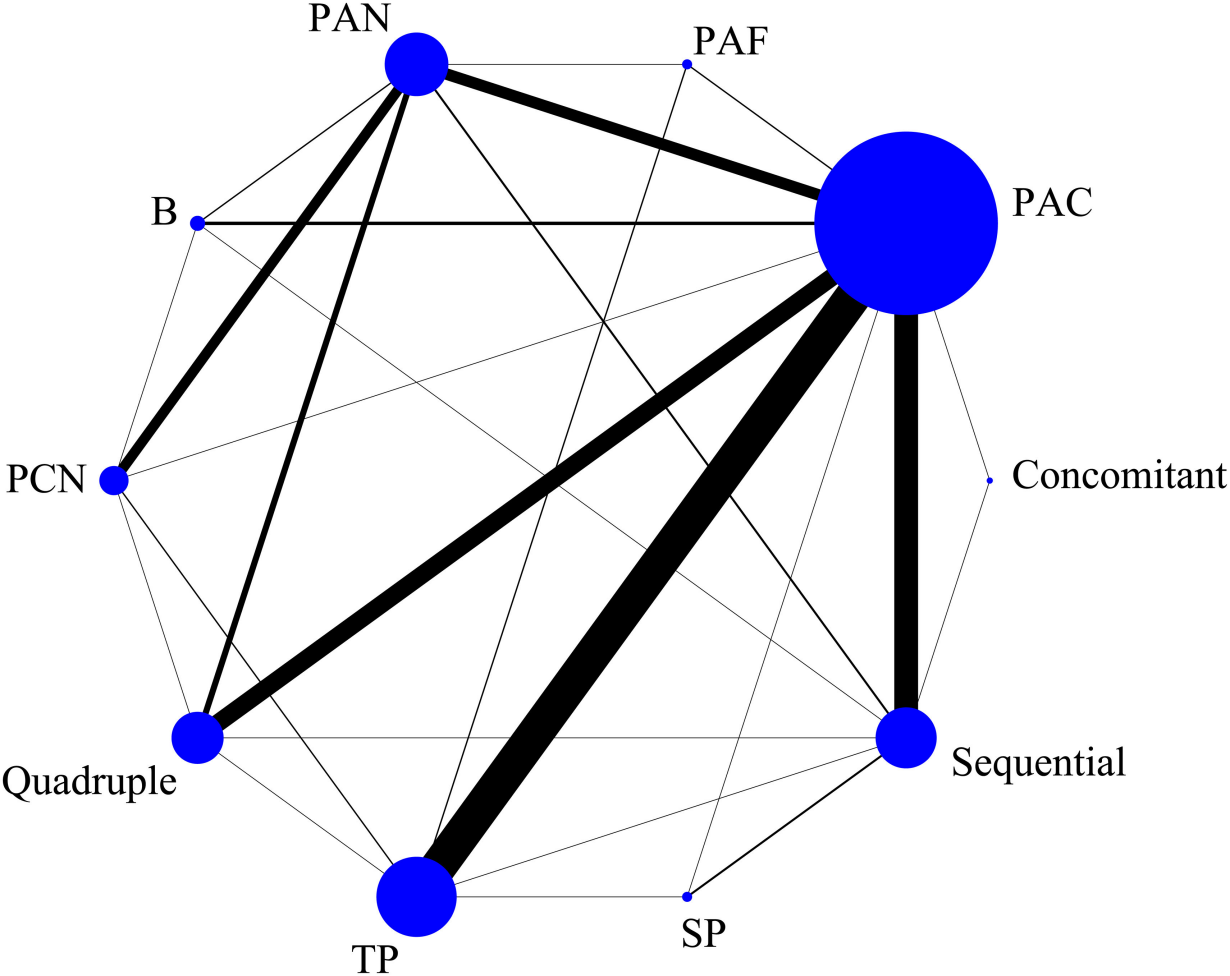
Network graph of eradication rate. The size of the blue node is positively correlated with the sample size involved in the intervention, and the thickness of the connection between two nodes represents the number of studies. Regimen labels: B, bismuth-containing triple therapy; Concomitant, concomitant therapy; PAC, PPI, amoxicillin and clindamycin; PAF, PPI, amoxicillin and furanes; PAN, PPI, amoxicillin and nitroimidazoles; PCN, PPI, clindamycin and nitroimidazoles; Quadruple, quadruple therapy; Sequential, sequential therapy; SP, sequential therapy with probiotics; TP, triple therapy with probiotics.

The eradication rate of *H. pylori* was used as the outcome measure. There are 26 direct comparisons, including closed loops, among the 10 treatment regimens in the network map. Each node in the map represents an intervention, and the lines between nodes represent a direct comparison between the two interventions. In addition, the node size represents the total sample size of the intervention, and the line thickness represents the number of studies directly compared. The results showed the standard triple therapy composed of proton-pump inhibitor (PPI), amoxicillin and clarithromycin was the most studied. The network map is shown in **Figure 3**.

### 3.5 Inconsistency test

**Figure 4:**
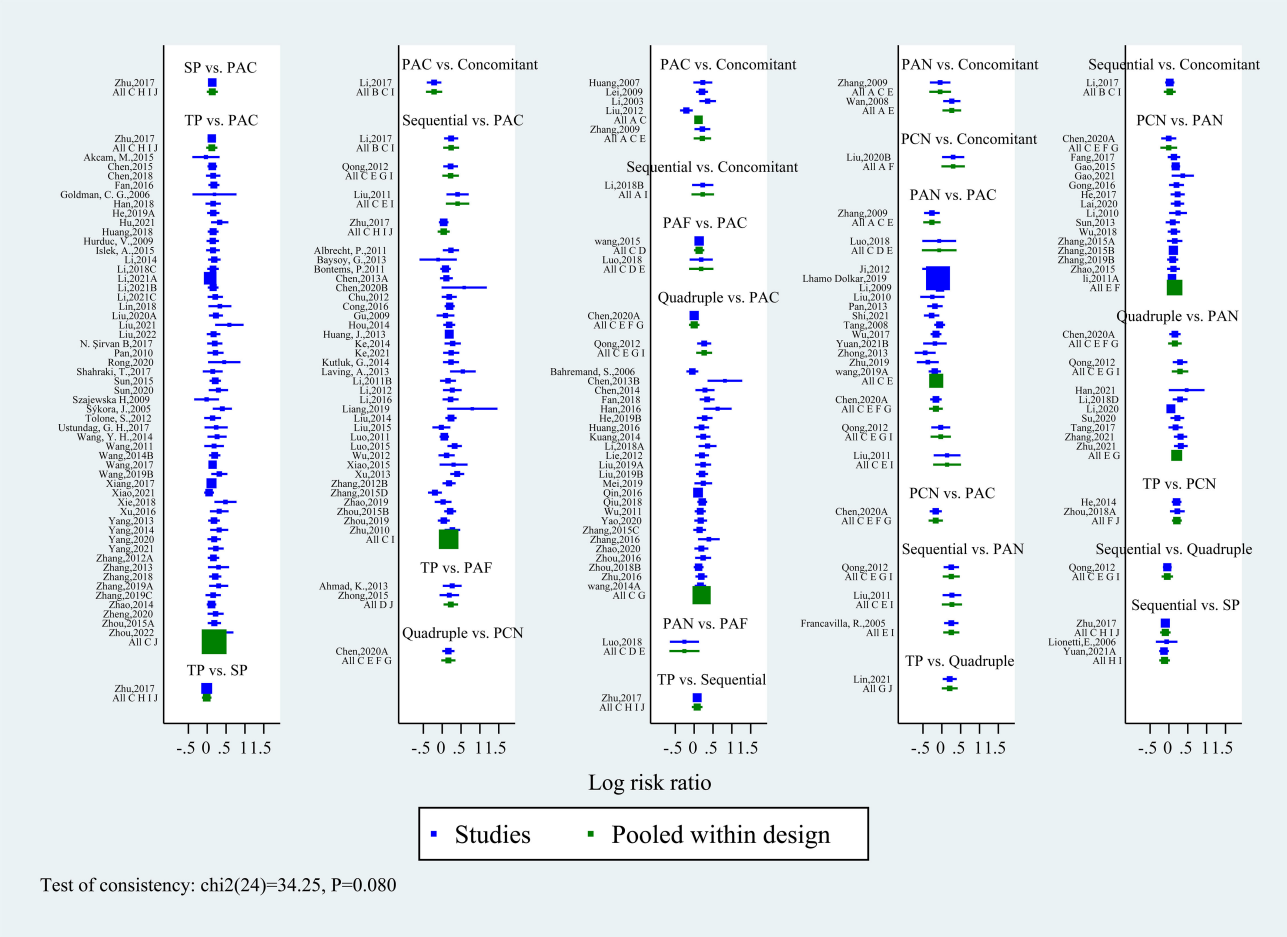
Forest plot of inconsistency test for 10 eradication regimens. The results show that the inconsistency is not significant (P>0.05). Regimen labels: B, bismuth-containing triple therapy; Concomitant, concomitant therapy; PAC, PPI, amoxicillin and clindamycin; PAF, PPI, amoxicillin and furanes; PAN, PPI, amoxicillin and nitroimidazoles; PCN, PPI, clindamycin and nitroimidazoles; Quadruple, quadruple therapy; Sequential, sequential therapy; SP, sequential therapy with probiotics; TP, triple therapy with probiotics.

Due to multiple closed-loop structures formed by 10 intervention nodes, the inconsistency test was carried out. The results showed that the inconsistency is not significant (P=0.08), which could be analyzed by the consistency model. The inconsistency test forest map is shown in **Figure 4**.

### 3.6 Overall cure rates and SUCRA probability ranking

**Figure 5:**
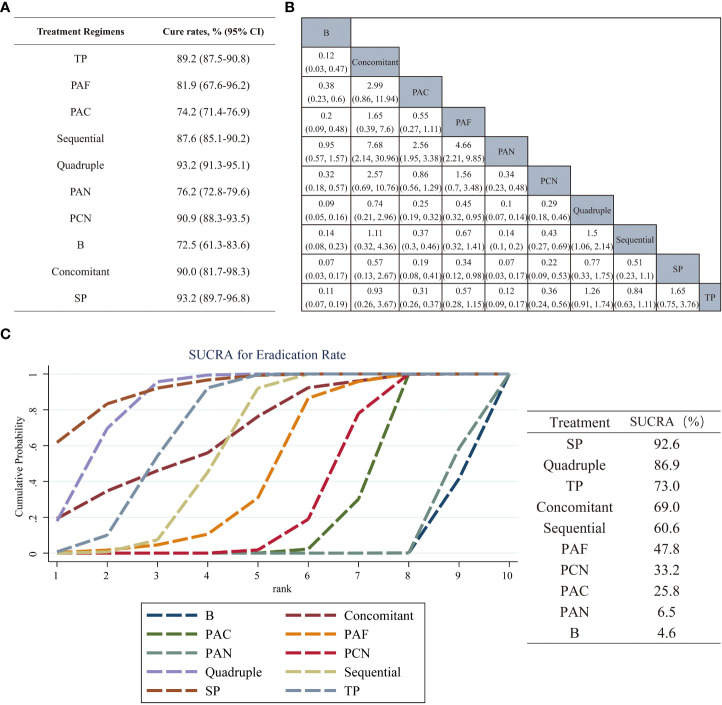
**(A)** Statistical results of the overall cure rates of 10 eradication regimens for *Helicobacter pylori* infection in children. **(B)** The comparison league table of 10 eradication programs. The values in the table are the effect sizes and 95% confidence intervals of the treatment measures in the column and the row. **(C)** The SUCRA was used to rank the efficacy of the 10 eradication regimens included. The larger the area under the curve, the better the therapeutic effect. Regimen labels: B, bismuth-containing triple therapy; Concomitant, concomitant therapy; PAC, PPI, amoxicillin and clindamycin; PAF, PPI, amoxicillin and furanes; PAN, PPI, amoxicillin and nitroimidazoles; PCN, PPI, clindamycin and nitroimidazoles; Quadruple, quadruple therapy; Sequential, sequential therapy; SP, sequential therapy with probiotics; TP, triple therapy with probiotics.

A Bayesian network meta-analysis of eradication rates was performed, and the overall cure rates for each intervention were calculated and compared with each other. **Figure 5A** shows the overall regimen cure rates of 10 regimens. The results signified that the eradication rates of SP, quadruple, concomitant and PCN therapies in children with *H. pylori* were at least 90%. The eradication rates of Sequential, TP and PAF therapies were more than 80%.

There was no difference in the efficacy in the pairwise comparison of PAF therapy, PCN therapy and PAC therapy. However, the effectiveness of SP therapy and quadruple therapy was significantly higher than that of the above three therapies. The comparisons of PCN therapy *vs* sequential therapy (OR, 0.43; 95% CI, 0.27–0.69), PCN therapy *vs* TP therapy (OR, 0.36; 95% CI, 0.24–0.56), PAC therapy *vs* sequential therapy (OR, 0.37; 95% CI, 0.3–0.46), PAC therapy *vs* TP therapy (OR, 0.31; 95% CI, 0.26–0.37) yielded significant results that sequential therapy and TP therapy were more effective. In contrast, the comparisons of sequential therapy *vs* PAF therapy, TP therapy *vs* PAF therapy revealed insignificant results. In terms of other comparisons, only the comparison between quadruple therapy and sequential therapy showed differences (OR, 1.5; 95% CI, 1.06 2.14). Pairwise comparison results are shown in **Figure 5B**.

The efficacy of the above 10 regimens was ranked, and the SUCRA values are shown in **Figure 5C**. The probability of different interventions was ranked as follows: SP (SUCRA 92.6%) > Quadruple (SUCRA 86.9%) > TP (SUCRA 73.0%) > Concomitant (SUCRA 69.0%) > Sequential (SUCRA 60.6%) > PAF (SUCRA 47.8%) > PCN (SUCRA 33.2%) > PAC (SUCRA 25.8%) > PAN (SUCRA 6.5%) > B (SUCRA 4.6%).

### 3.7 Safety outcomes

**Figure 6:**
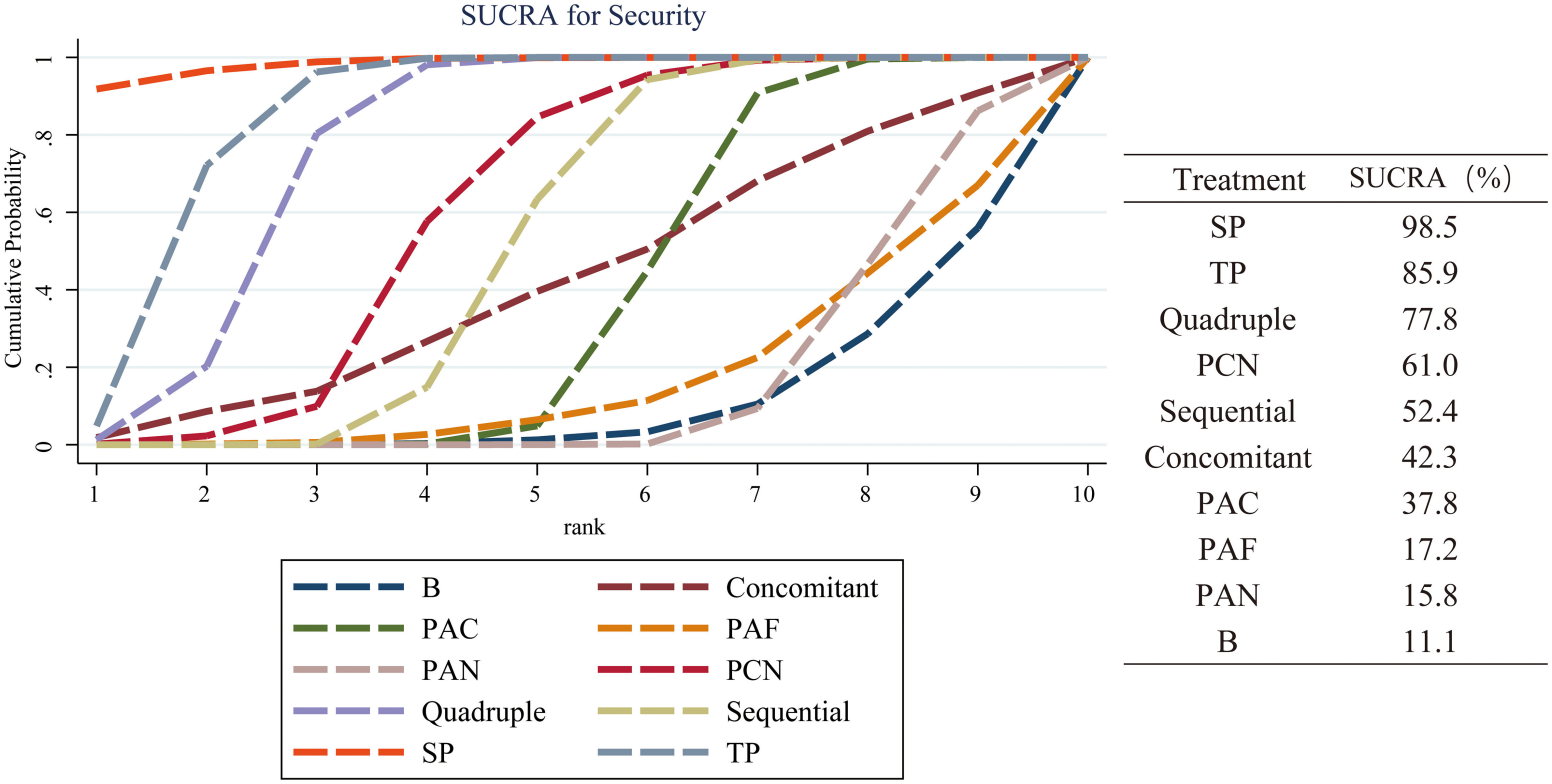
Safety evaluation of 10 regimens in children with *H. pylori* Infection. Taking total side effects rate as the safety index of the eradication scheme, the larger the SUCRA value and the area under the curve, the higher the security of the regimens. Regimen labels: B, bismuth-containing triple therapy; Concomitant, concomitant therapy; PAC, PPI, amoxicillin and clindamycin; PAF, PPI, amoxicillin and furanes; PAN, PPI, amoxicillin and nitroimidazoles; PCN, PPI, clindamycin and nitroimidazoles; Quadruple, quadruple therapy; Sequential, sequential therapy; SP, sequential therapy with probiotics; TP, triple therapy with probiotics.

Total side effects rate was used as the outcome measure to evaluate the safety of the 10 regimens. 116 articles reported the total cases of adverse reactions in each group, including constipation, diarrhea, rash, nausea, vomiting. All reactions were at a mild level, posing no influence on the therapies. The SUCRA is shown in **Figure 6**. The security ranking is as follows: SP (SUCRA 98.5%) > TP (SUCRA 85.9%) > Quadruple (SUCRA 77.8%) > PCN (SUCRA 61.0%) > Sequential (SUCRA 52.4%) > Concomitant (SUCRA 42.3%) > PAC (SUCRA 37.8%) > PAF (SUCRA 17.2%) > PAN (SUCRA 15.8%) > B (SUCRA 11.1%).

### 3.8 Subgroup analysis

**Figure 7:**
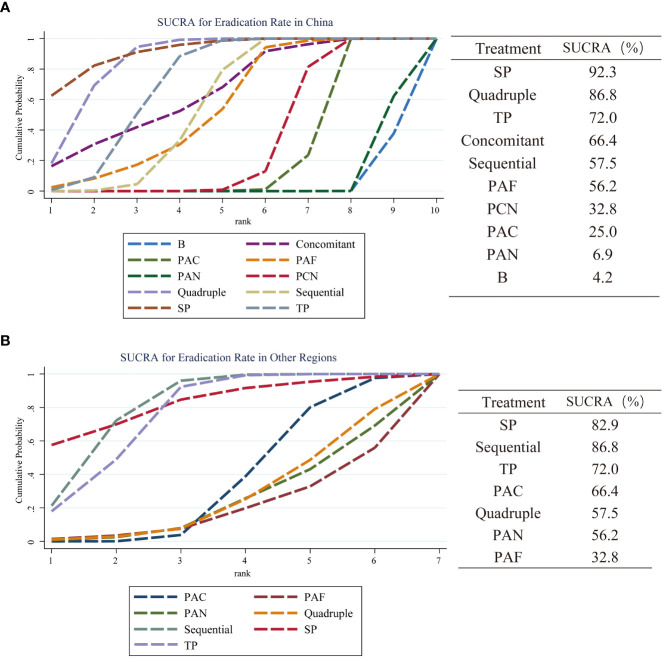
A subgroup analysis of the eradication rate of 10 treatment regimens in different regions. **(A)** SUCRA value of eradication rate and ranking of curative effect in China; **(B)** The performance of these results in other regions. Regimen labels: B, bismuth-containing triple therapy; Concomitant, concomitant therapy; PAC, PPI, amoxicillin and clindamycin; PAF, PPI, amoxicillin and furanes; PAN, PPI, amoxicillin and nitroimidazoles; PCN, PPI, clindamycin and nitroimidazoles; Quadruple, quadruple therapy; Sequential, sequential therapy; SP, sequential therapy with probiotics; TP, triple therapy with probiotics.

The literature included in this study has a clear regional distribution. Therefore, a subgroup analysis was performed according to the region where eligible studies were published (China and other regions) so as to identify the most effective eradication therapy for *H. pylori*. The results show that SP therapy (SUCRA 92.3%) has the best curative effect in China. The ranking of other options is as follows: Quadruple (SUCRA 86.8%), TP (SUCRA 72.0%), Concomitant (SUCRA 66.4%), Sequential (SUCRA 57.5%), PAF (SUCRA 56.2%), PCN (SUCRA 32.8%), PAC (SUCRA 25.0%), PAN (SUCRA 6.9%), B (SUCRA 4.2%). In other areas, only seven regimens are mentioned, and their curative effects are ranked as follows: SP (SUCRA 82.9%) > Sequential (SUCRA 81.5%) > TP (SUCRA 76.4%) > PAC (SUCRA 36.7%) > Quadruple (SUCRA 27.4%) > PAN (SUCRA 24.9%) > PAF (SUCRA 20.2%). The results of subgroup analysis are shown in **Figures 7A, B**.

### 3.9 Risk of bias assessment

**Figure 8:**
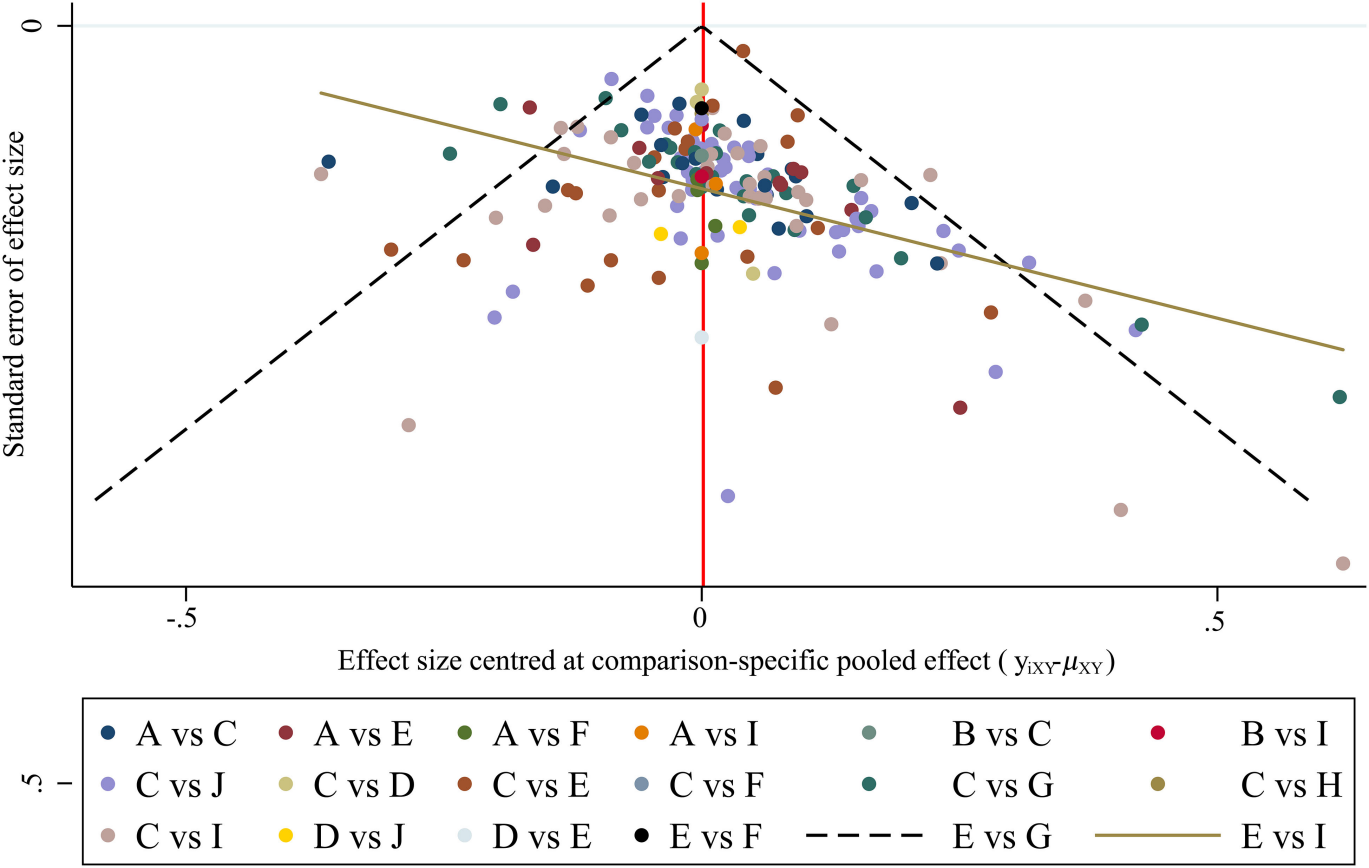
The funnel plots for assessing publication bias. The bias of the included literature is tested, and the result shows that research spots are symmetrically distributed. It means that the risk of the existence of publication bias or small-study effects in this network is low.

The bias funnel diagram is shown in **Figure 8**. As can be seen, most of the studies are at the top of the funnel chart, and roughly symmetrically distributed on both sides of the vertical line, so the possibility of publication bias and small-study effect is low.

## 4 Discussion

With the in-depth study of gastrointestinal diseases in recent years, *Helicobacter pylori* infection in children has been brought into focus, and the correlation between *H. pylori* infection and other systemic diseases has been gradually found (Zhang and Zhu, 2015). Due to unique characteristics of children’s growth and their own drug metabolism, *H. pylori* infection guidelines based on adults are not fully applicable. Since 2015, China, Occident, and Japan have successively published consensus and guidelines on the diagnosis and treatment of *Helicobacter pylori* infection in children, but there are differences in the recommended treatment regimens (Huang and Gong, 2015; Jones et al., 2017; Kato et al., 2020). Pediatric clinicians around the world try to use a variety of different solutions to treat *Helicobacter pylori* infection in children, but the eradication rate in some areas is not ideal. The present study summarized relevant published RCTs around the world, and compared and ranked the efficacy and safety of 10 reported eradication regimens for *H. pylori* infection in children. The overall results of these schemes show that SP therapy (SUCRA 92.6%) is the best. Quadruple therapy (SUCRA 86.9%), TP therapy (SUCRA 73.0%) and concomitant therapy (SUCRA 69.0%) are at least effective regimens. The results of the remaining regimens such as sequential therapy (SUCRA 60.6%), PAF therapy (SUCRA 47.8%), PCN therapy (SUCRA 33.2%), PAC therapy (SUCRA 25.8%), PAN therapy (SUCRA 6.5%) and B therapy (SUCRA 4.6%) are not satisfactory in this comparison. The ranking results of safety evaluation show that SP therapy and TP therapy have advantages in reducing side effects.

In the treatment of *H. pylori* infection in children, clinicians need to consider not only the eradication rate, but also medication compliance. Therefore, probiotics with rich tastes are widely accepted. Studies have shown that probiotics can inhibit *H. pylori* through a variety of immune and non-immune mechanisms, such as reducing the level of pro-inflammatory factors, increasing local IgA concentration, secreting antibacterial substances, and increasing gastric surface mucin to prevent the adhesion of pathogenic bacteria (Homan and Orel, 2015). A previous meta-analysis of 484 children found that triple therapy with Lactobacillus increased the eradication rate of *H. pylori* by 13% (Fang et al., 2019). The results of our analysis reveal that the addition of probiotics to sequential or triple therapy can increase the effectiveness.

Sequential therapy was initially proposed by Zullo (Zullo et al., 2000; Zullo et al., 2003), and in the subsequent prospective study, new sequential treatment was used and obtained fairly good results (eradication rate > 90%). However, the complicated medication mode of sequential therapy may lead to a decrease in children’s compliance. In contrast, the advantages of concomitant therapy on simplicity and easy operation make it successful in Korea (Lee et al., 2015). The results of the present study show that concomitant therapy merely ranks fourth (SUCRA69.0%). As a rescue treatment plan for adults after the failure of *Helicobacter pylori* eradication in the ACG guidelines (Chey et al., 2017), whether concomitant therapy can be used as the first treatment for children still needs careful consideration (Graham and Lee, 2015). The SUCRA values show that although the efficacy of bismuth-containing triple therapy (B, SUCRA 4.6%) is poor, the result of bismuth-containing quadruple therapy (Quadruple, SUCRA 86.9%) is unexpected, and we speculate that it is due to the addition of PPIs to this therapy. Sakurai et al. (2015) suggested that the eradication of *H. pylori* depended on a near-neutral gastric pH. PPIs have long been proved to effectively reduce gastric acid secretion and enhance antibiotic activity, which is conducive to the eradication of *H. pylori* (Miehlke et al., 1997). In the studies included in this analysis, metronidazole was used in almost all bismuth-containing triple therapy, but Graham pointed out that it needs to be combined with antisecretory drugs in the cases of metronidazole resistance (Graham et al., 2019). Judging from the results (eradication rate 93.2% vs 73.5%), quadruple therapy seems to be a good choice. However, it must be emphasized that: (1) Bismuth agents are unpalatable, such as colloidal bismuth citrate (De-Nol) has a very pungent ammonia smell, so it’s difficult for children to accept, which may eventually lead to insufficient dose or course of treatment and affect the effect. (2) The most suitable pH for bismuth to form bismuth salts and dissolve in the stomach is between 4 and 7. Concomitant administration of bismuth and antacids (PPIs such as omeprazole) will affect the formation of bismuth salts and reduce its absorption (Lambert and Midolo, 1997). (3) At present, the mechanism of bismuth to eradicate *H. pylori* is not completely clear. As a semi-metal or metalloid, bismuth salts have toxicity——although perhaps low toxicity——which may be one of the reasons for pediatricians to use it cautiously.

A Japanese study found that the prevalence of clarithromycin-resistant strains increased from 8.7% to 34.5% between 1997 and 2008, and the *H. pylori* eradication rate of triple therapy decreased from 90.6% to 74.8% (Sasaki et al., 2010). This phenomenon is still growing. Our pooled results also show that the overall eradication rate of PAC therapy is only 74%. In addition, the effect of PAN therapy is poor (eradication rate <80%), which is undoubtedly related to the rapid rise of drug resistance in this kind of drugs, especially metronidazole. The resistance rate of metronidazole in the Asia-Pacific region increased from 36% before 2000 to 45% in 2011-2015 (Savoldi et al., 2018). A new study in Chongqing, China found that the resistance rate of metronidazole in 156 children was as high as 88.4% (P = 0.0003) (Geng et al., 2022). This suggests that this therapy is likely to be abandoned by most clinicians. PAF therapy (SUCRA 47.8%) was initially ignored by us due to its low SUCRA values, but the statistical results exceeded our expectations because its overall eradication rate reached 81.9%. However, this is not a reason for us to give it a direct affirmation, because of its small sample size in this study.

In terms of safety outcomes, although not all researchers in eligible RCTs provided data of total side effects, we compared all regimens in the results of this study. According to the SUCRA values, the SP and TP therapies rank highest, suggesting that probiotics can significantly reduce the side effects of *H. pylori* eradication therapy. Daelemans (Daelemans et al., 2022) and Dargenio (Dargenio et al., 2022) supported our view in the latest review. Moreover, Wen (Wen et al., 2017) also found that some probiotics could effectively reduce specific side effects, which was helpful for clinical workers to adjust the medication in children.

The results of subgroup analysis show that SP therapy is the most effective regimen, and several different triple therapy regimens are the least effective regimens, whether in China or other regions. TP therapy ranks higher in both subgroups (surfaces under cumulative ranking 72.0% *vs* 76.4% respectively), so it may be an efficacious candidate for the treatment of *H. pylori* infection in children.

Generally speaking, the most important factors for successful eradication of *H. pylori* include resistance rate of antimicrobial, course of treatment, therapeutic regimen and patient compliance. It has been shown that eradication effects are associated with longer duration, regardless of first- or second-line treatment regimens (Yuan et al., 2013; Li et al., 2015; Muñoz et al., 2018; Yeo et al., 2018; Yeo et al., 2019). This reminds clinicians that it may be a better regimen to try to extend the dosing time rather than rush to change another one when the first formula has not achieved desired effects.

In short, the eradication rates of quadruple therapy, concomitant therapy and PCN therapy are at least 90% for empirical treatment of *H. pylori* infection in children. However, the results of standard triple therapy and Bismuth-containing triple therapy show that they are not completely reliable. The ranking results show that SP therapy is the most effective regimen in both China and other regions, and the ranking stability and approximately 90% eradication rate of TP therapy may be worthy of our attention. Safety evaluation shows that SP therapy and TP therapy can reduce side effects during the course of treatment in children.

The funnel plot suggests a low potential for bias, but there are still some limitations. On the one hand, only different regimens were compared, and no detailed distinction was made between different courses of treatment or doses of the same regimen. On the other hand, the number of literatures on probiotic-containing therapy accounts for a high proportion, which may have a certain impact on the analysis. In order to improve the stability and reliability of this study, in-depth research with large samples, multiple regions, and high-quality literature is still needed to optimize our results.

## 5 Conclusion

SP therapy is the most efficient and safest regimen for *H. pylori* infection in children, and TP therapy also has advantages in this network meta-analysis. Eradication rate of standard triple therapy is not exceptional.

## Data availability statement

The original contributions presented in the study are included in the article/**Supplementary Material**. Further inquiries can be directed to the corresponding author.

## Author contributions

ML and CZ, concept, design, research collection, data analysis, manuscript drafting. PZ, XZ, JS, data analysis, cross-checking, manuscript drafting, research collection. BY, concept, design, research collection, data analysis, manuscript review. The final submitted version has been confirmed by all authors. All authors contributed to the article and approved the submitted version.

## Glossary

**Table:**

**SUCRA**	Surface Under the Cumulative Ranking Curve
***H. pylori***	*Helicobacter pylori*
**NASPGHAN**	North American Society for Pediatric Gastroenterology, Hepatology, and Nutrition
**ESPGHAN**	European Society for Pediatric Gastroenterology, Hepatology, and Nutrition
**MALT**	mucosa-associated lymphoid tissue
**NMA**	network meta-analysis
**PPI**	Proton-pump inhibitor
**RCT**	Randomized Controlled Trial
**UBT**	urea breath test
**SAT**	ELISA stool antigen test
**OR**	odds ratio
**CI**	confidence intervals
**B**	Bismuth-containing triple therapy
**Concomitant**	Concomitant therapy
**PAC**	PPI, amoxicillin and clarithromycin
**PAF**	PPI, amoxicillin and nitrofuran drugs
**PAN**	PPI, amoxicillin and nitroimidazoles
**PCN**	PPI, clarithromycin and nitroimidazoles
**Quadruple**	Bismuth-containing quadruple therapy
**Sequential**	Sequential therapy
**SP**	Sequential therapy with probiotics
**TP**	Triple therapy with probiotics
