# Supplementary material for: Comparison of multiple treatment regimens in children with Helicobacter pylori infection: A network meta-analysis
Source: Front Cell Infect Microbiol. 2023 Feb 23;13:1068809. doi: 10.3389/fcimb.2023.1068809 (PMC9995679; doi:10.3389/fcimb.2023.1068809)
Supplement: Supplementary file 1 [file DataSheet_1.docx]

**Supplementary Table S1**

Search strategy

| **Databases** | **Search items** |
| --- | --- |
| Cochrane  Library | #1 MeSH descriptor: [Helicobacter pylori] explode all trees  #2 (Helicobacter pylori):ti,ab,kw OR (Helicobacter nemestrinae):ti,ab,kw OR (Campylobacter pylori):ti,ab,kw OR (Campylobacter pylori subsp. pylori):ti,ab,kw OR (Campylobacter pyloridis):ti,ab,kw  #3 #1 or #2  #4 MeSH descriptor: [Child] explode all trees  #5 (Child):ti,ab,kw OR (Children):ti,ab,kw  #6 #4 or #5  #7 MeSH descriptor: [Adolescent] explode all trees  #8 (Adolescent):ti,ab,kw OR (Adolescents):ti,ab,kw OR (Adolescence):ti,ab,kw OR (Teen*):ti,ab,kw OR (Youth):ti,ab,kw  #9 #7 or #8  #10 #6 or #9  #11 (randomized controlled trial):ti,ab,kw OR (controlled clinical trial):ti,ab,kw OR (double-blind):ti,ab,kw OR (single-blind):ti,ab,kw OR (Placebo):ti,ab,kw  #12 (Random*):ti,ab,kw OR (clinical trial*):ti,ab,kw OR (RCT):ti,ab,kw OR (RCTs):ti,ab,kw  #13 #11 or #12  #14 #3 and #10 and #13 |
| Pubmed | ("Helicobacter pylori"[MeSH Terms] OR ("Helicobacter pylori"[Title/Abstract] OR "helicobacter nemestrinae"[Title/Abstract] OR "campylobacter pylori"[Title/Abstract] OR ((("Helicobacter pylori"[MeSH Terms] OR ("Helicobacter"[All Fields] AND "pylori"[All Fields]) OR "Helicobacter pylori"[All Fields] OR ("Campylobacter"[All Fields] AND "pylori"[All Fields]) OR "campylobacter pylori"[All Fields]) AND ("subsp"[All Fields] OR "subsps"[All Fields])) AND "pylori"[Title/Abstract]) OR "campylobacter pyloridis"[Title/Abstract])) AND ("Child"[MeSH Terms] OR ("Child"[Title/Abstract] OR "Children"[Title/Abstract]) OR ("Adolescent"[MeSH Terms] OR ("Adolescent"[Title/Abstract] OR "Adolescents"[Title/Abstract] OR "Adolescence"[Title/Abstract] OR "teen*"[Title/Abstract] OR "Youth"[Title/Abstract] OR "Youths"[Title/Abstract]))) AND ("randomized controlled trial"[Title/Abstract] OR "controlled clinical trial"[Title/Abstract] OR "double-blind"[Title/Abstract] OR "single-blind"[Title/Abstract] OR "Placebo"[Title/Abstract] OR "random*"[Title/Abstract] OR "clinical trial*"[Title/Abstract] OR "RCT"[Title/Abstract] OR "RCTs"[Title/Abstract]) |
| EMBASE | #1. 'helicobacter pylori'/exp  #2. 'helicobacter pylori':ab,ti OR 'helicobacter nemestrinae':ab,ti OR 'campylobacter pylori':ab,ti OR 'campylobacter pylori subsp. pylori':ab,ti OR 'campylobacter pyloridis':ab,ti  #3. #1 OR #2  #4. 'child'/exp  #5. child:ab,ti OR children:ab,ti OR adolescent:ab,ti OR adolescents:ab,ti OR adolescence:ab,ti OR teen*:ab,ti OR youth:ab,ti OR youths:ab,ti  #6. #4 OR #5  #7. 'randomized controlled trial':ab,ti OR 'controlled clinical trial':ab,ti OR 'double blind':ab,ti OR 'single blind':ab,ti OR placebo:ab,ti OR random*:ab,ti OR 'clinical trial*':ab,ti OR rct:ab,ti OR rcts:ab,ti  #8. #3 AND #6  #9. #7 AND #8 |
| Web of science | #1  Helicobacter pylori (Topic) or Helicobacter nemestrinae (Topic) or Campylobacter pylori (Topic) or Campylobacter pylori subsp. pylori (Topic) or Campylobacter pyloridis (Topic)  #2 Child (Topic) or Children (Topic) or Adolescent (Topic) or Adolescents (Topic) or Adolescence (Topic) or Teen* (Topic) or Youth (Topic) and Youths (Topic)  #3  randomized controlled trial (Topic) or controlled clinical trial (Topic) or double-blind (Topic) or single-blind (Topic) or Placebo (Topic) or Random* (Topic) or clinical trial* (Topic) or RCT (Topic) or RCTs (Topic)  #4  #1 AND #2 AND #3 |
| CNKI | (篇关摘=幽门螺杆菌 + 幽门螺旋菌 + 幽门螺旋杆菌 + 螺旋菌) AND (篇关摘=儿童 + 幼儿 + 小儿 + 未成年人) AND (篇关摘=随机) |
| CBM | ("幽门螺杆菌"[常用字段:智能] OR "幽门螺旋菌"[常用字段:智能] OR "幽门螺旋杆菌"[常用字段:智能] OR "螺旋菌"[常用字段:智能]) AND ("儿童"[常用字段:智能] OR "幼儿"[常用字段:智能] OR "小儿"[常用字段:智能] OR "未成年人"[常用字段:智能]) AND ("随机"[常用字段:智能]) |
| VIP | (U=幽门螺杆菌+幽门螺旋菌+幽门螺杆菌+螺旋菌) AND (U=儿童+幼儿+小儿+未成年人) AND (U=随机) |
| Wanfang  Databases | 全部:(幽门螺杆菌 or 幽门螺旋菌 or 幽门螺杆菌 or 螺旋菌) and 主题:(儿童 or 幼儿 or 小儿 or 未成年人) and 全部:(随机) |

**Supplementary Table S2**

Baseline characteristics of the included studies in this Network Meta-Analysis

| Study ID | Country | Study design | Number of patients per arm | Regimens compared |
| --- | --- | --- | --- | --- |
| Ahmad, K.,2013^[17]^ | Iran | RCT-2 arm | 33/33 | TP/ PAF |
| Akcam, M.,2015^[18]^ | Turkey | RCT-2 arm | 27/29 | TP/ PAC |
| Albrecht, P.,2011^[19]^ | Poland | RCT-2 arm | 52/51 | Sequential / PAC |
| Zhu,2021^[20]^ | China | RCT-2 arm | 49/49 | Quadruple / PAN |
| Bahremand, S.,2006^[21]^ | Iran | RCT-2 arm | 50/50 | Quadruple / PAC |
| N Şirvan B ,2017^[22]^ | Japan | RCT-2 arm | 50/50 | TP/ PAC |
| Baysoy, G.,2013^[23]^ | Turkey | RCT-2 arm | 37/24 | Sequential / PAC |
| Bontems, P.2011^[24]^ | Belgium | RCT-2 arm | 77/73 | Sequential / PAC |
| Chen,2013A ^[25]^ | China | RCT-2 arm | 35/32 | Sequential / PAC |
| Chen,2013B^[26]^ | China | RCT-2 arm | 56/50 | Quadruple / PAC |
| Chen,2014^[27]^ | China | RCT-2 arm | 48/48 | Quadruple / PAC |
| Chen,2015^[28]^ | China | RCT-2 arm | 120/120 | TP / PAC |
| Chen,2018^[29]^ | China | RCT-2 arm | 46/46 | TP / PAC |
| Chen,2020A ^[30]^ | China | RCT-4 arm | 50/51/45/52 | PAC / PAN / PCN / Quadruple |
| Chen,2020B^[31]^ | China | RCT-2 arm | 35/33 | Sequential / PAC |
| Chu,2012^[32]^ | China | RCT-2 arm | 43/35 | Sequential / PAC |
| Cong,2016^[33]^ | China | RCT-2 arm | 63/63 | Sequential / PAC |
| Fan,2016^[34]^ | China | RCT-2 arm | 75/75 | TP/ PAC |
| Fan,2018^[35]^ | China | RCT-2 arm | 50/50 | Quadruple / PAC |
| Fang,2017^[36]^ | China | RCT-2 arm | 43/43 | PAN / PCN |
| Francavilla, R.,2005^[37]^ | Italy | RCT-2 arm | 37/37 | Sequential / PAN |
| Gao,2015^[38]^ | China | RCT-2 arm | 105/105 | PAN / PCN |
| Gao,2021^[39]^ | China | RCT-2 arm | 28/28 | PAN / PCN |
| Goldman, C. G.,2006^[40]^ | Argentina | RCT-2 arm | 33/32 | TP/ PAC |
| Gong,2016^[41]^ | China | RCT-2 arm | 35/35 | PAN / PAC |
| Gu,2009^[42]^ | China | RCT-2 arm | 28/22 | Sequential / PAC |
| Han,2016^[43]^ | China | RCT-2 arm | 30/30 | Quadruple / PAC |
| Han,2018^[44]^ | China | RCT-2 arm | 58/58 | TP/ PAC |
| Han,2021^[45]^ | China | RCT-2 arm | 40/40 | Quadruple / PAN |
| He,2014^[46]^ | China | RCT-2 arm | 80/80 | TP/ PCN |
| He,2017^[47]^ | China | RCT-2 arm | 41/42 | PAN / PCN |
| He,2019A ^[48]^ | China | RCT-2 arm | 60/60 | TP / PAC |
| He,2019B^[49]^ | China | RCT-2 arm | 44/44 | Quadruple / PAC |
| Hou,2014^[50]^ | China | RCT-2 arm | 65/65 | Sequential / PAC |
| Hu,2021^[51]^ | China | RCT-2 arm | 42/42 | TP/ PAC |
| Huang,2007^[52]^ | China | RCT-2 arm | 40/40 | B / PAC |
| Huang,2016^[53]^ | China | RCT-2 arm | 43/43 | Quadruple / PAC |
| Huang,2018^[54]^ | China | RCT-2 arm | 42/42 | TP/ PAC |
| Huang, J.,2013^[55]^ | China | RCT-2 arm | 107/211 | Sequential / PAC |
| Hurduc, V.,2009^[56]^ | Romania | RCT-2 arm | 48/42 | TP / PAC |
| Islek, A.,2015^[57]^ | Turkey | RCT-2 arm | 41/39 | TP/ PAC |
| Ji,2012^[58]^ | China | RCT-2 arm | 34/28 | PAN / PAC |
| Ke,2014^[59]^ | China | RCT-2 arm | 63/57 | Sequential / PAC |
| Ke,2021^[60]^ | China | RCT-2 arm | 43/42 | Sequential / PAC |
| Kuang,2014^[61]^ | China | RCT-2 arm | 45/45 | Quadruple / PAC |
| Kutluk, G.,2014^[62]^ | Turkey | RCT-2 arm | 32/46 | Sequential / PAC |
| Lhamo Dolkar,2019^[63]^ | China | RCT-2 arm | 541/541 | PAN / PAC |
| Lai,2020^[64]^ | China | RCT-2 arm | 34/34 | PAN / PCN |
| Laving, A.,2013^[65]^ | Kenya | RCT-2 arm | 26/45 | Sequential / PAC |
| Lei,2009^[66]^ | China | RCT-2 arm | 60/80 | B / PAC |
| Li,2003^[67]^ | China | RCT-2 arm | 60/60 | B / PAC |
| Li,2009^[68]^ | China | RCT-2 arm | 45/45 | PAN / PAC |
| Li,2010^[69]^ | China | RCT-2 arm | 30/30 | PAN / PCN |
| Li,2011A^[70]^ | China | RCT-2 arm | 100/100 | PAN / PCN |
| Li,2011B^[71]^ | China | RCT-2 arm | 34/32 | Sequential / PAC |
| Li,2012^[72]^ | China | RCT-2 arm | 34/33 | Sequential / PAC |
| Li,2014^[73]^ | China | RCT-2 arm | 82/82 | TP/ PAC |
| Li,2016^[74]^ | China | RCT-2 arm | 38/38 | Sequential / PAC |
| Li,2017^[75]^ | China | RCT-3 arm | 50/50/51 | Sequential / Concomitant / PAC |
| Li,2018A^[76]^ | China | RCT-2 arm | 59/59 | Quadruple / PAC |
| Li,2018B^[77]^ | China | RCT-2 arm | 29/30 | B / Sequential |
| Li,2018C ^[78]^ | China | RCT-2 arm | 44/44 | TP/ PAC |
| Li,2018D^[79]^ | China | RCT-2 arm | 40/40 | Quadruple / PAN |
| Li,2020^[80]^ | China | RCT-2 arm | 40/40 | Quadruple / PAN |
| Li,2021A^[81]^ | China | RCT-2 arm | 150/150 | TP/ PAC |
| Li,2021B^[82]^ | China | RCT-2 arm | 50/50 | TP PAC |
| Li,2021C^[83]^ | China | RCT-2 arm | 73/72 | TP / PAC |
| Liang,2019^[84]^ | China | RCT-2 arm | 44/44 | Sequential / PAC |
| Lie,2012^[85]^ | China | RCT-2 arm | 33/33 | Quadruple / PAC |
| Lin,2018^[86]^ | China | RCT-2 arm | 45/45 | TP/ PAC |
| Lin,2021^[87]^ | China | RCT-2 arm | 39/39 | TP/ Quadruple |
| Lionetti, E.,2006^[88]^ | Italy | RCT-2 arm | 20/20 | SP / Sequential |
| Liu,2010^[89]^ | China | RCT-2 arm | 20/20 | PAN / PAC |
| Liu,2011^[90]^ | China | RCT-3 arm | 34/33/33 | Sequential / PAN / PAC |
| Liu,2012^[91]^ | China | RCT-2 arm | 45/45 | B / PAC |
| Zhu,2019^[92]^ | China | RCT-2 arm | 32/32 | PAN / PAC |
| Liu,2014^[93]^ | China | RCT-2 arm | 78/78 | Sequential / PAC |
| Liu,2015^[94]^ | China | RCT-2 arm | 47/42 | Sequential / PAC |
| Liu,2019A ^[95]^ | China | RCT-2 arm | 35/35 | Quadruple / PAC |
| Liu,2019B^[96]^ | China | RCT-2 arm | 69/69 | Quadruple / PAC |
| Liu,2020A ^[97]^ | China | RCT-2 arm | 74/74 | TP/ PAC |
| Liu,2020B^[98]^ | China | RCT-2 arm | 31/31 | B / PCN |
| Liu,2021^[99]^ | China | RCT-2 arm | 30/30 | TP/ PAC |
| Liu,2022^[100]^ | China | RCT-2 arm | 50/50 | TP/ PAC |
| Luo,2011^[101]^ | China | RCT-2 arm | 60/60 | Sequential / PAC |
| Luo,2015^[102]^ | China | RCT-2 arm | 45/47 | Sequential / PAC |
| Luo,2018^[103]^ | China | RCT-3 arm | 29/15/21 | PAF / PAN / PAC |
| Mei,2019^[104]^ | China | RCT-2 arm | 31/30 | Quadruple / PAC |
| Pan,2010^[105]^ | China | RCT-2 arm | 40/35 | TP / PAC |
| Pan,2013^[106]^ | China | RCT-2 arm | 32/32 | PAN / PAC |
| Qin,2016^[107]^ | China | RCT-2 arm | 60/60 | Quadruple / PAC |
| Qiu,2018^[108]^ | China | RCT-2 arm | 63/63 | Quadruple / PAC |
| Rong,2012^[109]^ | China | RCT-4 arm | 50/50/50/50 | Quadruple / Sequential / PAN / PAC |
| Rong,2020^[110]^ | China | RCT-2 arm | 29/29 | TP / PAC |
| Shahraki, T.,2017^[111]^ | Argentina | RCT-2 arm | 25/25 | TP/ PAC |
| Shi,2021^[112]^ | China | RCT-2 arm | 40/40 | PAN / PAC |
| Zhu,2017^[113]^ | China | RCT-4 arm | 109/102/105/100 | SP / Sequential / TP / PAC |
| Su,2020^[114]^ | China | RCT-2 arm | 37/36 | Quadruple / PAN |
| Sun,2013^[115]^ | China | RCT-2 arm | 34/34 | PAN / PCN |
| Sun,2015^[116]^ | China | RCT-2 arm | 48/48 | TP / PAC |
| Sun,2020^[117]^ | China | RCT-2 arm | 40/40 | TP / PAC |
| Sýkora, J.,2005^[118]^ | Czech Republic | RCT-2 arm | 36/44 | TP / PAC |
| Szajewska H,2009^[119]^ | Poland | RCT-2 arm | 34/32 | TP / PAC |
| Tang,2008^[120]^ | China | RCT-2 arm | 56/100 | PAN / PAC |
| Tang,2017^[121]^ | China | RCT-2 arm | 40/40 | Quadruple / PAN |
| Tolone, S.,2012^[122]^ | Italy | RCT-2 arm | 34/34 | TP / PAC |
| Ustundag, G. H.,2017^[123]^ | Turkey | RCT-2 arm | 33/31 | TP / PAC |
| Wan,2008^[124]^ | China | RCT-2 arm | 50/50 | B / PAN |
| Wang,2011^[125]^ | China | RCT-2 arm | 27/27 | TP / PAC |
| Wang,2014A^[126]^ | China | RCT-2 arm | 40/40 | Quadruple / PAC |
| Wang,2014B^[127]^ | China | RCT-2 arm | 52/52 | TP / PAC |
| Wang,2015^[128]^ | China | RCT-2 arm | 100/100 | PAF / PAC |
| Wang,2017^[129]^ | China | RCT-2 arm | 96/102 | TP / PAC |
| Wang,2019A^[130]^ | China | RCT-2 arm | 50/50 | PAN / PAC |
| Wang,2019B ^[131]^ | China | RCT-2 arm | 42/42 | TP / PAC |
| Wang, Y. H.,2014^[132]^ | China | RCT-2 arm | 43/45 | TP / PAC |
| Wu,2011^[133]^ | China | RCT-2 arm | 41/41 | Quadruple / PAC |
| Wu,2012^[134]^ | China | RCT-2 arm | 30/30 | Sequential / PAC |
| Wu,2017^[135]^ | China | RCT-2 arm | 60/60 | PAN / PAC |
| Wu,2018^[136]^ | China | RCT-2 arm | 43/43 | PAN / PCN |
| Xiang,2017^[137]^ | China | RCT-2 arm | 250/250 | TP / PAC |
| Xiao,2015^[138]^ | China | RCT-2 arm | 30/30 | Sequential / PAC |
| Xiao,2021^[139]^ | China | RCT-2 arm | 45/45 | TP / PAC |
| Xie,2018^[140]^ | China | RCT-2 arm | 45/42 | TP / PAC |
| Xu,2013^[141]^ | China | RCT-2 arm | 75/75 | Sequential / PAC |
| Xu,2016^[142]^ | China | RCT-2 arm | 62/58 | TP / PAC |
| Yang,2013^[143]^ | China | RCT-2 arm | 93/93 | TP / PAC |
| Yang,2014^[144]^ | China | RCT-2 arm | 52/68 | TP / PAC |
| Yang,2020^[145]^ | China | RCT-2 arm | 69/69 | TP / PAC |
| Yang,2021^[146]^ | China | RCT-2 arm | 50/50 | TP / PAC |
| Yao,2020^[147]^ | China | RCT-2 arm | 55/55 | Quadruple / PAC |
| Yuan,2021A^[148]^ | China | RCT-2 arm | 60/60 | SP / Sequential |
| Yuan,2021B ^[149]^ | China | RCT-2 arm | 66/65 | PAN / PAC |
| Zhang,2009^[150]^ | China | RCT-3 arm | 33/40/35 | B / PAN / PAC |
| Zhang,2012A ^[151]^ | China | RCT-2 arm | 38/40 | TP / PAC |
| Zhang,2012B^[152]^ | China | RCT-2 arm | 45/45 | Sequential / PAC |
| Zhang,2013^[153]^ | China | RCT-2 arm | 30/30 | TP / PAC |
| Zhang,2015A^[154]^ | China | RCT-2 arm | 30/30 | PAN / PCN |
| Zhang,2015B^[155]^ | China | RCT-2 arm | 84/84 | PAN / PCN |
| Zhang,2015C^[156]^ | China | RCT-2 arm | 48/48 | Quadruple / PAC |
| Zhang,2015D ^[157]^ | China | RCT-2 arm | 43/43 | Sequential / PAC |
| Zhang,2016^[158]^ | China | RCT-2 arm | 39/39 | Quadruple / PAC |
| Zhang,2018^[159]^ | China | RCT-2 arm | 49/49 | TP / PAC |
| Zhang,2019A^[160]^ | China | RCT-2 arm | 42/40 | TP / PAC |
| Zhang,2019B^[161]^ | China | RCT-2 arm | 45/45 | PCN / PAN |
| Zhang,2019C ^[162]^ | China | RCT-2 arm | 58/58 | TP / PAC |
| Zhang,2021^[163]^ | China | RCT-2 arm | 50/50 | Quadruple / PAN |
| Zhao,2014^[164]^ | China | RCT-2 arm | 120/120 | TP / PAC |
| Zhao,2015^[165]^ | China | RCT-2 arm | 49/49 | PAN / PCN |
| Zhao,2019^[166]^ | China | RCT-2 arm | 31/33 | Sequential / PAC |
| Zhao,2020^[167]^ | China | RCT-2 arm | 43/43 | Quadruple / PAC |
| Zheng,2020^[168]^ | China | RCT-2 arm | 45/45 | TP / PAC |
| Zhong,2013^[169]^ | China | RCT-2 arm | 49/49 | PAN / PAC |
| Zhong,2015^[170]^ | China | RCT-2 arm | 34/34 | TP / PAF |
| Zhou,2015A^[171]^ | China | RCT-2 arm | 58/58 | TP / PAC |
| Zhou,2015B^[172]^ | China | RCT-2 arm | 69/69 | Sequential / PAC |
| Zhou,2016^[173]^ | China | RCT-2 arm | 35/35 | Quadruple / PAC |
| Zhou,2018A^[174]^ | China | RCT-2 arm | 35/35 | Quadruple / PAC |
| Zhou,2018B ^[175]^ | China | RCT-2 arm | 50/50 | TP / PCN |
| Zhou,2019^[176]^ | China | RCT-2 arm | 90/90 | Sequential / PAC |
| Zhou,2022^[177]^ | China | RCT-2 arm | 60/60 | TP / PAC |
| Zhu,2010^[178]^ | China | RCT-2 arm | 82/41 | Sequential / PAC |
| Zhu,2016^[179]^ | China | RCT-2 arm | 48/48 | Quadruple / PAC |
